# Tetrandrine Modulates Rheb-mTOR Signaling-Mediated Selective Autophagy and Protects Pulmonary Fibrosis

**DOI:** 10.3389/fphar.2021.739220

**Published:** 2021-11-22

**Authors:** Yuanyuan Liu, Wenshan Zhong, Jinming Zhang, Weimou Chen, Ye lu, Yujie Qiao, Zhaojin Zeng, Haohua Huang, Shaoxi Cai, Hangming Dong

**Affiliations:** Department of Respiratory and Critical Care Medicine, Chronic Airways Diseases Laboratory, Nanfang Hospital, Southern Medical University, Guangzhou, China

**Keywords:** lung fibrosis, tetrandrine, autophagy, mTOR, COL-I

## Abstract

Idiopathic pulmonary fibrosis is a progressive fatal disease characterized by interstitial remodeling, with high lethality and a lack of effective medical therapies. Tetrandrine has been proposed to present anti-fibrotic effects, but the efficacy and mechanisms have not been systematically evaluated. We sought to study the potential therapeutic effects and mechanisms of tetrandrine against lung fibrosis. The anti-fibrotic effects of tetrandrine were evaluated in bleomycin-induced mouse models and TGF-β1-stimulated murine lung fibroblasts. We performed Chromatin Immunoprecipitation (ChIP), Immunoprecipitation (IP), and mRFP-GFP-MAP1LC3B adenovirus construct to investigate the novel mechanisms of tetrandrine-induced autophagy. Tetrandrine decreased TGF-β1-induced expression of α-smooth muscle actin, fibronectin, vimentin, and type 1 collagen and proliferation in fibroblasts. Tetrandrine restored TGF-β1-induced impaired autophagy flux, accompanied by enhanced interaction of SQSTM1 and MAP1LC3-Ⅱ. ChIP studies revealed that tetrandrine induced autophagy via increasing binding of NRF2 and SQSTM1 promoter. Furthermore, tetrandrine inhibited TGF-β1-induced phosphorylation of mTOR by reducing activation of Rheb. *In vivo* tetrandrine suppressed the bleomycin-induced expression of fibrotic markers and improved pulmonary function. Our data suggest that protective effect of tetrandrine against lung fibrosis might be through promoting Rheb-mTOR and NRF2-SQSTM1 mediated autophagy. Tetrandrine may thus be potentially employed as a novel therapeutic medicine against IPF.

## Introduction

Idiopathic pulmonary fibrosis is a prototype of chronic, progressive, and fibrotic lung disease, characterized by repetitive injury of the lung epithelium, activation and proliferation of (myo) fibroblasts, and accumulation of extracellular matrix (King et al., 2011; Hutchinson et al., 2015). Despite this high unmet clinical need, only two anti-fibrotics drugs, Pirfenidone and nintedanib, have been approved to be effective in slowing down the decline of lung function in IPF patients. However, neither agent stops the progression of IPF (King et al., 2014; Richeldi et al., 2014). Thus, there is a tremendous interest in investigating the pathological mechanisms underlying IPF in order to identify novel therapies.

Tetrandrine (TET) is a low-toxicity drug extracted from the plant Stephania tetrandra S. Moore (Fenfangji) of the menispermaceae (Bhagya and Chandrashekar 2016). Previous studies have reported that TET could exert anti-fibrotic effects on multiple organs, primarily by interfering with autophagy (Wang et al., 2015). In addition, TET had also been identified as an effective inducer of autophagy (Liu et al., 2017). However, the mechanism underlying the protective effects of TET on lung fibrosis remains unclear.

Autophagy is the process in which cells degrade internal constituents for the maintenance of cellular homeostasis (Barth et al., 2010) and is known to participate in removing ubiquitinated proteins (Ciani et al., 2003). SQSTM1/p62 is a signaling hub and a critical selective autophagy receptor (Lippai and Low 2014). Intriguingly, autophagy deficit, a common feature of many diseases, plays an important role in various fibrotic diseases, including liver, kidney, and pulmonary fibrosis (Patel et al., 2012; Araya et al., 2013). Transforming growth factor β1 (TGF-β1) is one of the major profibrotic cytokines in fibrosis diseases that could inhibit autophagy during myofibroblast differentiation in lung fibroblasts (Sosulski et al., 2015). Furthermore, autophagy may contribute to the degradation of COL1/collagen I to alleviate fibrosis (Moscat and Diaz-Meco 2009; Sosulski et al., 2015).

The purpose of the current study was to analyze the anti-fibrotic effect of TET on TGF-β1-induced fibroblast transdifferentiation and bleomycin-induced murine lung fibrosis. We demonstrate that TET therapy decreases fibrotic markers *in vitro* and *in vivo*. These protective effects of TET on pulmonary fibrosis are associated with activation of autophagy through promoting NRF2-SQSTM1 axis and Rheb-mTOR signaling. Taken together, our findings provide important proof-of-concept evidence that activation autophagy induced by TET could be used as a novel pharmacological approach for treatment of human IPF.

## Materials and Methods

### Culture of Lung Fibroblasts

Primary mouse lung fibroblasts (pMLFs) were isolated from the lungs obtained from C57BL/6J mice and maintained in DMEM supplemented with 10% FBS and penicillin–streptomycin. pMLFs were isolated using a method described previously (Bueno et al., 2015). Briefly, mouse lungs were minced into 1–2 mm^3^ pieces and incubated in calcium- and magnesium-free Hanks’ balanced salt solution (HBSS) containing 1,000 U/ml collagenase A for 30 min, and after washing with HBSS, then add 0.25% trypsin-EDTA for 20 min at 37°C with shaking. The dissociated cells were centrifuged and cultured in DMEM supplemented with 10% FBS for 1 h, and then adherent fibroblasts were rinsed with HBSS and cultured in DMEM supplemented with 10% FBS and penicillin–streptomycin. More than 95% of the cells were morphologically fibroblasts and stained with vimentin, and no cells were stained with CD45. The fibroblasts were used between culture passages 3 and 6.

Human lung fibroblast line IMR90 was purchased from American Type Culture Collection (Manassas, VA). IMR90 were maintained in DMEM supplemented with 10% FBS, 100 units/mL penicillin, and 100 g/ml streptomycin in 5% CO_2_ and 95% humidity at 37°C.

### Cell Viability Assay

Cell viability was measured by a cell counting Kit-8 (CCK8) assay (Dojindo, Japan). Cells were seeded in 200 μl of growth medium at a density of 8 × 10^3^ cells per well in 96-well plates. Cells were treated with or without TET for 24 h. Following the manufacturer’s recommendations, 10 μl CCK-8 solution was added per well for 2 h before the end of incubation at 37°C. Cell viability was measured at an absorbance of 450 nm.

### Transfection of siRNA and Plasmids

Different siRNA oligos were obtained from Gene Pharma (Shanghai, China), and the sequences were listed in Supplementary Table S1. Plasmids for Rheb overexpression were purchased from Hanbio (Shanghai, China) and the sequence is shown in Supplementary Table S1. Cells were transfected with the appropriate siRNA oligos or plasmid with Lipofectamine 3,000 (Invitrogen, CA, United States), according to the manufacturer’s protocol. After 48 h, cells were further stimulated with different reagents. The successfully transfected clones were confirmed by western blotting.

### Tandem Fluorescent- mRFP-GFP-MAP1LC3B-Adenovirus Transduction of pMLFs

pMLFs were transfected with a tandem fluorescent-mRFP-GFP-MAP1LC3B-adenovirus (HanBio, HB-AP2100001) that expresses a specific marker of autophagosome formation to detect autophagy, according to the manufacturer’s instructions (Hariharan et al., 2011). With this tandem construct, autophagosomes and autolysosomes are labelled with yellow (mRFP and GFP) and red (mRFP only) signals, respectively. Five fields were chosen from 3 different cell preparations. GFP- and mRFP-expressing spots, which were indicated by fluorescent puncta and DAPI-stained nuclei, were counted manually. The number of spots per cell was determined by dividing the total number of spots by the number of nuclei in each field.

### Immunofluorescence Assay

The immunofluorescence assay was performed as described previously (Ohashi et al., 2015). Cells were seeded on a confocal dish for 24 h. After treatment, cells were fixed with 4% paraformaldehyde for 15 min in phosphate-buffered saline (PBS), followed by permeabilization for 10 min with Triton X-100–containing buffer. Antibodies was used for immunofluorescence assays. Then, cells were incubated for 12 h at 4°C with corresponding antibodies. Next, cells were washed with PBS and incubated with Alexa Fluor 594 anti-Rat (1:200) and Alexa Fluor 488 anti-mouse (1:200) (Life Technologies, CA, United States) at room temperature for 2 h afterwards. The nuclei of the cells were stained with DAPI (Invitrogen). Images were taken on an Olympus FV1000 Confocal Laser Scanning Microscope (Tokyo, Japan). The following antibodies were used: MAP1LC3B (CST, 2775s, 83506S), SQSTM1 (proteintech, 18420-1-AP, 66184-1-AP), Anti-alpha smooth muscle Actin (Abcam, ab7817), Cleaved caspase-3 (CST, 9664s), ubiquitin (Proteintech, 10201-2-AP), Phospho-4E-BP1 (Thr37/46) (236B4) (CST, 2855T), LAMP2 (Abcam, Ab13524), Collagen1 (Affinitiy, AF7001).

### Network Pharmacology and Bioinformatics Analysis

Pulmonary fibrosis-related genes were obtained from Comparative Toxicogenomics database (CTD, http://ctdbase.org/) and DisGeNET (a database of gene-disease associations, https://www.disgenet.org/) using the term “pulmonary fibrosis,” followed by filtering with the term “*Homo sapiens.*” Potential targets of TET were obtained from the Swiss target prediction (http://www.swisstargetprediction.ch/) and pubchem (https://pubchem.ncbi.nlm.nih.gov/). A total of 74 potential human targets were obtained and the official gene names were obtained from Uniprot (http://www.uniprot.org/) by confining the species to “*Homo sapiens.*” Subsequently, various ID forms of the targets were transformed into UniProt IDs. The Search Tool for the Retrieval of Interacting Genes (STRING) database (https://string-db.org/) supplies each predicted PPI information as well as the data which have actually been experimentally confirmed. Then, we seek out the intersection through Cytoscape-Bisogenet (3.7.2) and screen out the top 10 key genes. The functional pathways of TET related to pulmonary fibrosis were analyzed using the Kyoto Encyclopedia of Genes and Genomes (KEGG) pathway and gene ontology (GO) enrichment evaluation based upon the database for annotation, Visualization and Integrated Discovery (DAVID) version 6.8 (https://david.ncifcrf.gov/). *p*-value was calculated in these two enrichment analyses, and *p* < 0.05 suggests the enrichment degree was statistically significant and the pathway results would certainly be necessary functional mechanisms of pulmonary fibrosis.

### Western blotting and Co-Immunoprecipitation

Total cell lysates were obtained using the Total Protein Extraction Kit (KeyGen Biotech, China) according to the manufacturer’s instructions. Proteins were subjected to SDS-PAGE, transferred to PVDF membranes, and probed with various primary antibodies and LICOR: fluorescence-labeled secondary antibodies (#926-68071, 926-32,210). Blots were visualized using a LICOR Odyssey fluorescent imaging system (LICOR Biotechnology) finally. For co-immunoprecipitation, protein extracts from MLFs were incubated with indicated primary antibody overnight at 4°C. The immune-complexes were cleared with Protein A/G Magnetic Beads (Thermo Scientific). Input lysates were run simultaneously with the IP samples on 10% polyacrylamide gels and visualized with LICOR Odyssey Scanner. Antibodies used were: anti-β-actin (Proteintech, 66009-1-Ig), anti-phospho-mTOR (Ser2448; Cell Signaling Technology, 5536S), anti-Phospho-P70 (Thr389; Cell Signaling Technology, 9236S), anti-Phospho-4E-BP1 (Thr37/46; Cell Signaling Technology, 2855T), anti-phospho-smad3 (Ser423/425; Cell Signaling Technology, 9520T), anti-phospho-smad2 (Ser465/Ser467; Cell Signaling Technology, 18338T), anti-MAP1LC3-I/II (Cell Signaling Technology, 2775S), anti-cleaved caspase3 (Cell Signaling Technology, 9664S), anti-α-SMA (Abcam, ab32575), anti-fibronectin (Abcam, ab2413), Anti-Rheb (Santa Cruz, sc-271509), anti-SQSTM1 (Proteintech, 18420-1-AP), anti-4E-BP1 (Proteintech, 60246-Ig), anti-p70(S6K) (Proteintech, 14485AP), anti-mTOR (Proteintech, 20657-1-AP), anti-ATG7 (Proteintech, 10088-2-AP), anti-vimentin (Proteintech, 10366-1-AP), anti-IgG (Proteintech, B900610), anti-smad3 (Proteintech, 25494-1-AP), anti-smad 2 (Proteintech, 12570-1-AP), anti-Col-I (Affinity, AF7001) Ubiquitin (Proteintech, 10201-2-AP).

### Chromatin Immunoprecipitation Assay

The immunoprecipitation (ChIP) assay in MLFs was performed using the SimpleChIP Enzymatic Chromatin IP Kit (CST, 9003) according to the manufacturer’s protocol. Approximately 4  ×  10^6^ cells were used for each immunoprecipitation. Chromatin was immunoprecipitated with the immunoglobulin G (CST, #2729; as a negative control) or NRF2 (Genetex, #GTX103322). In all, 10% total DNA was used for input evaluation. DNA enrichment in the ChIP samples was determined by reverse transcription and semi-quantitative PCR (RT-PCR) with PrimeScript™ RT reagent Kit with gDNA Eraser (Takara, China) and a LightCycler 96 Instrument (Roche) following the manufacturer’s protocol. PCR products of immunoprecipitated and input samples were analyzed on a 2% agarose gel. Specific primer sets for the SQSTM1 locus are shown as follow: SQSTM1_P1 forward: ATT​CTG​CCC​TGC​ATG​TCT​T, reverse: GCC​TTC​TAG​GTA​TGG​TCC​TTT​C; SQSTM1_P2 forward: TGG​CCG​AGC​CTT​GAA​TTA​G, reverse: GCA​CCT​GCC​TAG​TAT​GTG​TT.

### RNA Extraction and Quantitative Real-Time PCR

Total RNA was extracted from cells with the TRIzol reagent (Takara, Dalian, China). The quantity and quality of RNA were determined using a Nanodrop 2000 spectrophotometer (Thermo Fisher Scientific), and then RNA was reverse transcribed using iScript™ cDNA synthesis kit (Biorad; 1708890) according to the manufacturer’s instructions. The qPCR was performed using TaqMan^®^ universal PCR Master Mix (Fisher Scientific; 4304437) on a StepOnePlus™ Real-Time PCR System (Thermo Fisher Scientific) in 20 μl reaction. Relative mRNA levels were calculated using the 2^−ΔΔCT^ method normalized against β-actin in each reaction. The following primers were used, Actb, forward: GTG​ACG​TTG​ACA​TCC​GTA​AAG​A, reverse: GCC​GGA​CTC​ATC​GTA​CTC​C; SQSTM1, forward: ATG​TGG​AAC​ATG​GAG​GGA​AGA, reverse: GGA​GTT​CAC​CTG​TAG​ATG​GGT; Rheb, forward: GGT​CTG​TGG​GAA​AGT​CCT​CAT, reverse: GGT​GAA​CGT​GTT​CTC​TAT​GGT​T.

### Bleomycin-Induced Lung Fibrosis Model and Treatment With Tetrandrine

All animal experiments were conducted according to Southern Medical University Animal Welfare, and research protocols were approved by the Institutional Animal Care and Use Committee of Southern Medical University. Mice were housed four mice per cage in a specific pathogen-free room with a 12 h light/dark schedule at 25°C ± 1°C and were fed an autoclaved chow diet and water ad libitum. An established mammalian model of idiopathic pulmonary fibrosis (IPF) was utilized as described in previous publications. Six- to eight-week-old C57BL/6 female mice (16–20 g) were anesthetized (2,2,2-tribromoethanol, Sigma-Aldrich) and then injected intratracheally with prepared bleomycin sulfate (5 U kg^−1^) (Hanhui pharmaceuticals co., LTD.) in sterile PBS (volume was varied between 80 and 100 μl depending on the body weight). Control mice were injected with 100 μl of sterile PBS. Body weights were monitored throughout each study. Each experimental group consisted of at least five animals.

To quantitate fibrosis during longitudinal studies, lungs were harvested at 21 days after bleomycin instillation and assayed as described below. To test the prevention efficacy of TET in our modeling of IPF, TET (20 mg kg^−1^, Sigma-Aldrich) or vehicle (equal volume of 0.1% sodium carboxymethyl cellulose) were administered by intraperitoneal injection at every other day after bleomycin administration, and the mice were sacrificed at day 21. For delayed therapy studies, induction of IPF was initiated as described above, and TET (20 mg kg^−1^ or 40 mg kg^−1^) was intraperitoneally injected every other day beginning on day 8. Lungs were harvested on day 21 and assayed as described below (day 0 was taken as the day of bleomycin administration).

### Pulmonary Function Assay

At endpoint, at least five mice from each group were anesthetized with 2,2,2-tribromoethanol in saline, tracheotomized below the larynx, and intubated with a tracheal cannula. After the surgery, the mice were placed inside the plethysmographic chamber and the cannula was connected to the machine. Pulmonary function was measured by pulmonary function test system (BUXCO, United States). The system’s software automatically records and displays the pulmonary function parameters.

### Immunoanalysis and Histopathology

Formalin-fixed, paraffin-embedded (FFPE) tissue blocks obtained from mouse models were sectioned at 5 mm. For immunohistochemistry, the FFPE unstained slides were deparaffinized through standard methods. Paraffin embedded sections of mouse lung tissue were pretreated in citrate buffer pH6 for 20 min for antigen retrieval. Sections were then incubated with corresponding antibodies. For murine fibrosis assessment, FFPE lung tissue blocks were sectioned at 5 mm and subjected to haematoxylin and eosin and Masson’s trichrome staining. Sections were reviewed by a blinded pathologist and approximately half of the specimens were scored by a second blinded pathologist to confirm agreement. Specimens were scored according to an eight-tier, modified Ashcroft scale (Hübner et al., 2008).

### Hydroxyproline Assay

Lung hydroxyproline content was analyzed using a hydroxyproline assay kit (#A030-2, Nanjing Jianchen Bioengineering Institute, China) according to the manufacturer’s instructions.

## Materials

Dimethyl sulfoxide (DMSO) and chloroquine (CQ) were obtained from Sigma-Aldrich (St. Louis, MO, United States). TGF-β1 were purchased from R&D Systems, Inc. (Minneapolis, MN, United States). 3-Methyladenine (3 MA) and MHY1485 were purchased from MedChemExpress (United States).

### Data and Statistical Analysis

All data were analyzed blinded and presented as scatter plots showing each single data point representing the number of independent values and means ± SEM using bars and whiskers. Group sizes in the animal experiment were n ≥ 5. The number of included data per group was mentioned in the figure legends. After testing values for normal distribution using Kolmogorov-Smirnov test, data were analyzed by: (1) Two-tailed t tests for comparison of two group means, using nonparametric analysis (Wilcoxon or Mann-Whitney) for n = 5; (2) One-way analysis of variance (ANOVA) followed by Dunnett’s multiple comparisons test was used for multiple comparisons to control for concentration of TET dose effects; (3) Two-way ANOVA followed by Tukey post hoc tests for three or more group means with two factors. Non-parametric data were analyzed by Dunnett’s multiple comparison test. Post hoc tests were run only if F achieved *p* < 0.05 and there was no significant variance in homogeneity. Differences between group means were considered statistically significant at the level of *p* < 0.05. Statistical analysis was performed using GraphPad Prism eight for Windows software (version 8.4.2, GraphPad software Inc, San Diego, CA, United States). The data and statistical analysis comply with the recommendations on experimental design and analysis in pharmacology (Curtis, Alexander et al., 2018).

## Results

### Tetrandrine Inhibited TGF-β1-Induced Myofibroblasts Differentiation, ECM Deposition, and Proliferation

**FIGURE 1:**
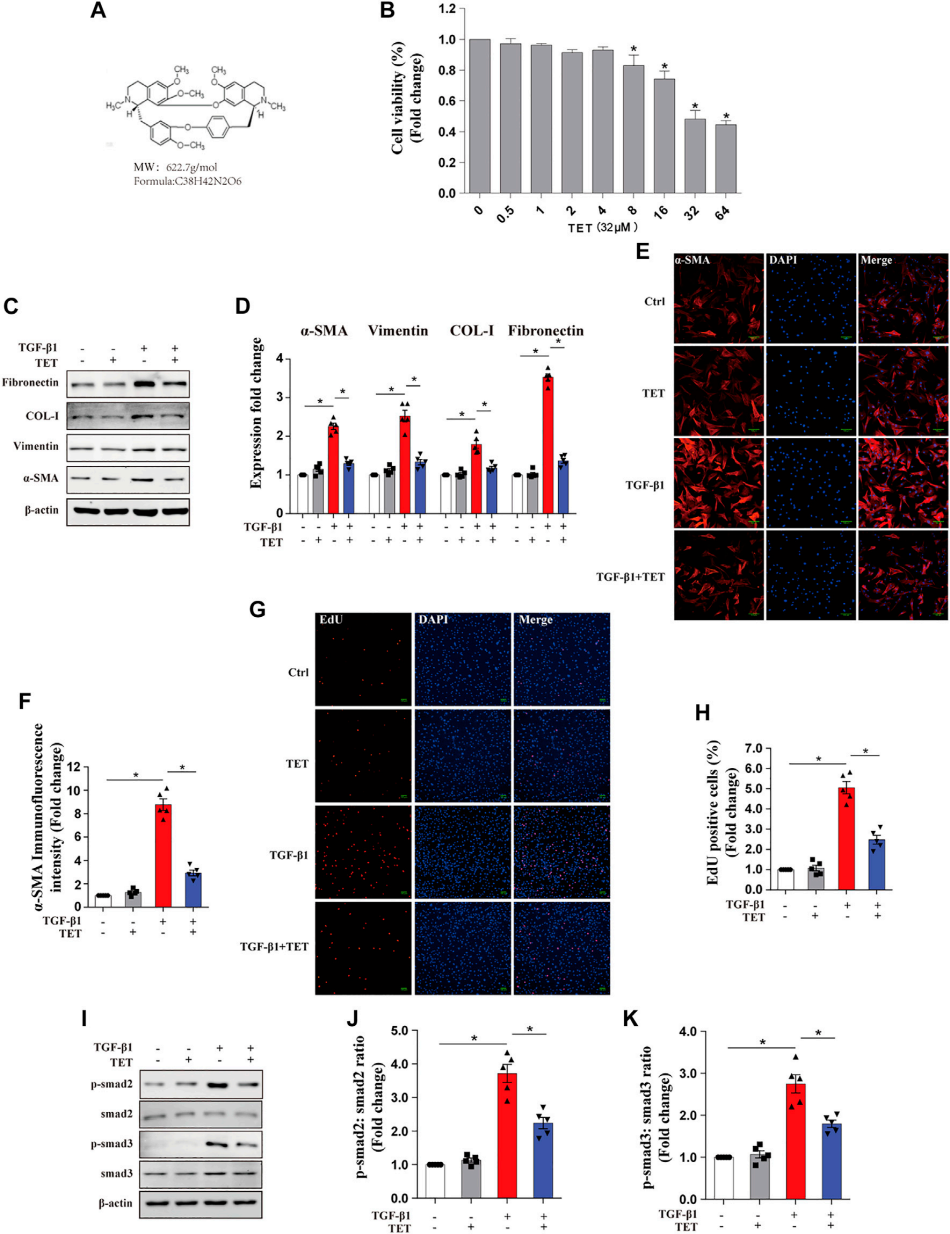
Tetrandrine suppresses TGF-β1-induced myofibroblasts differentiation, proliferation, and ECM deposition in primary MLFs. **(A)** Chemical structure of TET. Its Molecular formation is C_38_H_42_N_2_O_6_. **(B)** Primary MLFs were incubated with various indicated concentrations of TET for 24 h and subjected to CCK8 assay to assess cell viability. One-way ANOVA with Dunnett’s multiple comparison test: *p < 0.05 comparison to TET = 0 μM group value: *p < 0.05. **(C,D)** Primary MLFs were pretreated with DMSO or TET (4 μM) for 1 h and stimulated with or without TGF-β1 (10 ng/ml) for 24 h. Representative immunoblot analysis **(C)** and quantitative analysis **(D)** show the expression of ECM deposition (fibronectin, COL-I), myofibroblasts transdifferentiation (vimentin, α-SMA). **(E,F)** Representative Immunofluorescence monitored by confocal microscopy **(E)** and quantitative analysis **(F)** show the expression of α-SMA (red) in Primary MLFs. Blue staining indicates nuclei Scale bars: 100 μM. **(G,H)** Representative Immunofluorescence **(G)** and quantitative analysis **(H)** of *EdU* -positive cell proportion show the proliferation marker (*EdU:* 5-ehtynal-2′-deoxyuridine) in myofibroblast. Scale bars: 100 μM. **(I-K)** Representative immunoblot analysis **(I)** and quantitative analysis **(J,K)** show the expression of TGF-β/smad pathway markers (p-smad2, smad2, p-smad3, smad3) in primary MLFs. *p* values were determined by two-way ANOVA with Tukey’s multiple comparison test (n = 5): *p < 0.05.

Fibroblast differentiation is critical pathogenesis processes in IPF (Baek et al., 2012). Previous studies have reported anti-fibrotic effects of TET (chemical structure shown in Figure 1A) in multiple organs (Yin et al., 2007; Teng et al., 2015)*.* However, whether TET is capable of deactivating differentiated myofibroblasts and resolving fibrosis is not well known. We confirmed that 50% inhibition of cell growth was not achieved until TET concentration at 32 μM in primary mouse lung fibroblasts (pMLFs) or 32 μM in IMR90 cells (Figure 1B, Supplementary Figure S1A). Western blots and Immunofluorescence (IF) revealed that the expressions of fibronectin, type 1 collagen, vimentin, and α-SMA were all increased in pMLFs and IMR90 cells by stimulation with TGF-β1, indicating fibroblast differentiation and excessive matrix protein production. The effects of TGF-β1 were blocked by administration of TET (Figures 1C–F, Supplementary Figures S1B–E). Moreover, Numbers of *EdU* (+) cells after TGF-β1 exposure were significantly reduced by TET treatment for pMLFs (Figures 1G,H). TGF-β1-induced Smad2/3 activation indicated by Smad2/3 phosphorylation was blocked by TET in pMLFs (Figures 1I–K). Taken together, these results suggest that TET suppresses fibroblast differentiation and proliferation.

### Potential Target Genes and Network Analysis of TET Treatment for Pulmonary Fibrosis

**FIGURE 2:**
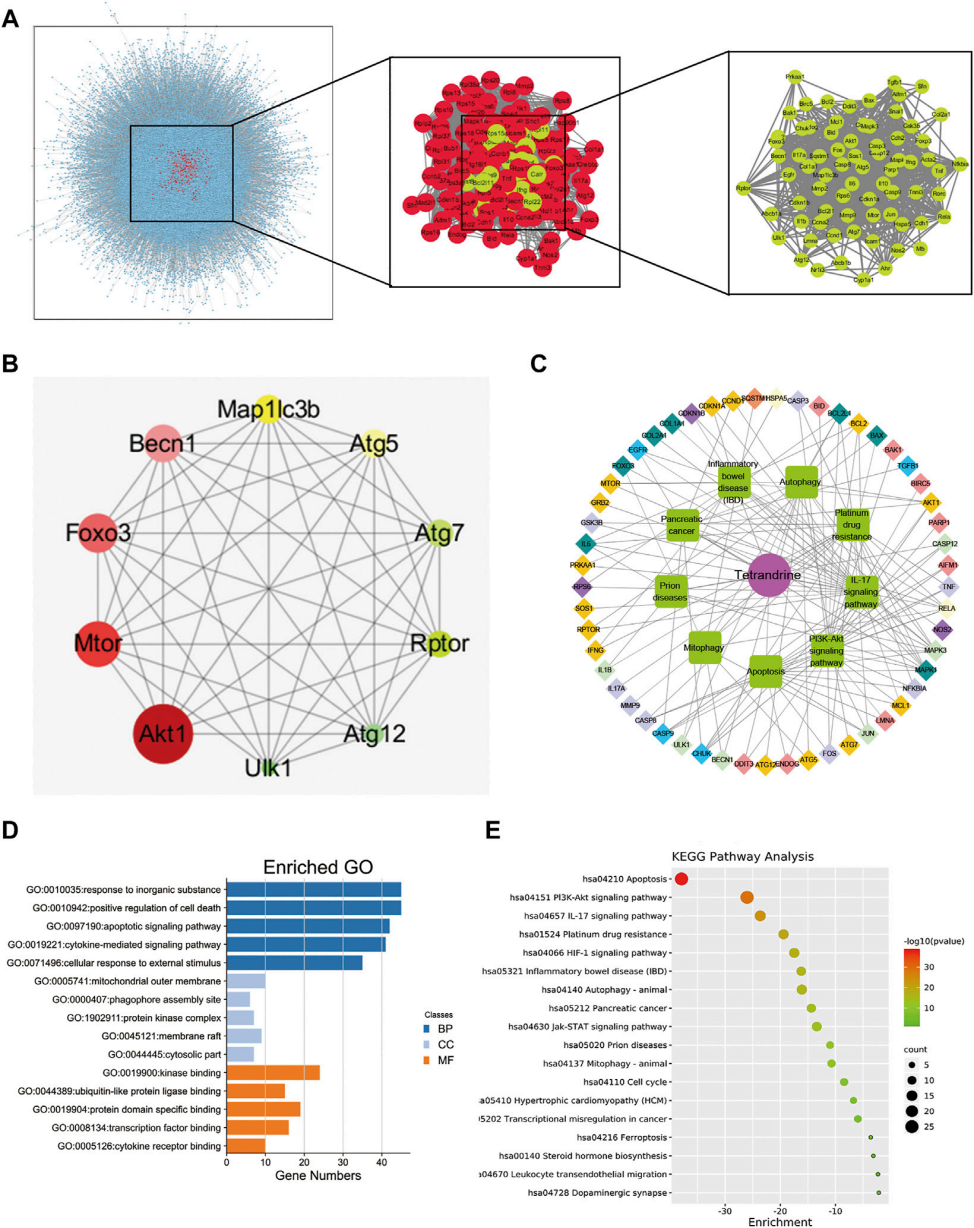
Analysis of TET’s targets on pulmonary fibrosis based on network pharmacology. **(A)** protein—protein interaction (PPI) networks of active ingredients of TET for the treatment of pulmonary fibrosis. Each node represents the relevant genes, the edge means line thickness indicates the strength of data support. **(B)** Hub top 10 genes in PPI network, the lighter the color, the higher the score; the larger the diameter, the higher the score. **(C)** Representative networks assembled by predicted TET targets. Diamonds in various colors nodes represent the targets for TET identified through target mapping. Green nodes represent signaling pathways or processes. Targets are connected with pathways or processes with inner circle in the corresponding color. **(D)** The GO analysis was discovered with the top five enriched conditions in the biological process (BP), cell component (CC), and molecular function (MF) categories. **(E)** KEGG pathways of target genes.

To elucidate the potential anti-fibrotic mechanisms of TET, we conducted an integrated systems pharmacology approach as previously described (Klionsky et al., 2007). We compared the respective gene-expression profiles of TET (from LINCS) to that of IPF and identified 74 target genes in IPF lungs visualized using Cytoscape (Figure 2A). Ten hub genes (AKT1, mTOR, Foxo3, Beclin1, MAP1LC3B, ATG5, ATG7, Rptor, ATG12, and ULK1) were identified in protein-protein interaction (PPI) networks (Figure 2B). We performed GO and KEGG pathway enrichment analysis of above gene sets using Cytoscape. The top enriched biological process is cell metabolism and the top cell component is membrane composition (Figure 2D). Interestingly, PI3K/AKT signaling, apoptosis, and autophagy are the top enriched gene ontology terms in KEGG pathway enrichment analysis (Figures 2C–E). Thus, the above data suggest that TET may relieve pulmonary fibrosis by regulating apoptosis and/or autophagy pathway.

### TET Restored TGF-β1-Induced Impaired Autophagy in MLFs

**FIGURE 3:**
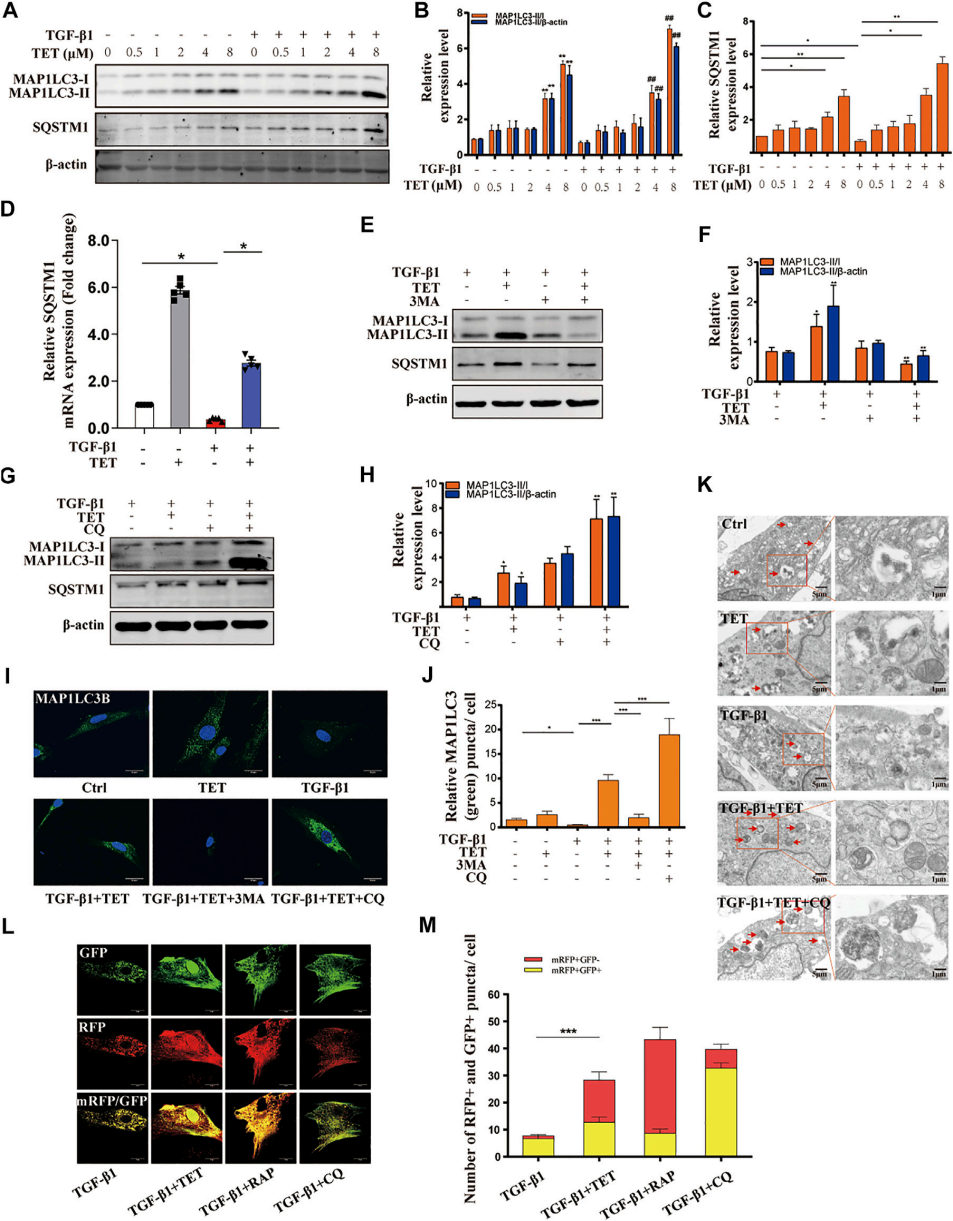
TET restores TGF-β1-induced impaired autophagy in primary MLFs. **(A-C)** Primary MLFs were pretreated with TET for indicated concentrations for 1 h and then subsequently stimulated with or without TGF-β1 (10 ng/ml) for 24 h. Representative immunoblot analysis **(A)** and quantitative analysis **(B–C)** showed the expression of autophagy (MAP1LC3-I/II, SQSTM1) markers and β-actin (loading control). Values in bar graph are presented as means ± SEM (n = 5). Two-way ANOVA: *p < 0.05, **p < 0.01, versus the control group; #*p* < 0.05, ##*p* < 0.01, versus the TGF-β1 group. **(D)** Primary MLFs were pretreated with DMSO or TET (4 μM) for 1 h and stimulated with or without TGF-β1 (10 ng/ml) for 24 h. Representative of qPCR analysis showed the mRNA expression of SQSTM1. **(E,F)** Primary MLFs were exposed to TGF-β1 (10 ng/ml) for 24 h. In some experimental groups, cells were treated with TET (4 μM), 3-methyladenine (3 MA: 500 nM). Immunoblot assays **(E)** and densitometric analysis **(F)** showed the expression of autophagic markers. **(G,H)** Primary MLFs were treated with TGF-β1 (10 ng/ml) in the presence/absence of TET (4 μM) or chloroquine (CQ: 20 μM). The protein sample were collected 24 h after the treatment. The levels of autophagic markers were examined with immunoblotting **(G)**. Relative levels of autophagic markers were determined by densitometry and normalized to β-actin levels (H). **(I,J)** Primary MLFs were treated with TGF-β1 (0 or 10 ng/ml) for 24 h in the presence/absence of TET (4 μM), 3 MA (500 nM), or CQ (20 μM). Representative images of immunofluorescence monitored by confocal microscopy **(I)** and quantification **(J)** showed the expression of autophagic marker (MAP1LC3B). Green staining is MAP1LC3B, blue staining indicates nuclei. Scale bars: 20 μm. **(K)** The transmission electron microscopy images showed numerous double-membraned cytoplasmic vacuolation (arrows). Scale bars: left panels, 5 μm; right panels, 1 m. **(L,M)** Primary MLFs were transfected with mRFP-GFP-LC3B plasmids for 48 h and treated with TGF-β1 (10 ng/ml) for 24 h in the presence/absence of TET (4 μM), rapamycin (RAP: 50 nM) or CQ (20 μM). Representative immunofluorescent images showed mRFP (green), GFP (red) and merged mRFP and GFP (yellow) puncta **(L)**. Scale bars: 10 µm. Quantification of red (mRFP + GFP−) and yellow (mRFP + GFP+) puncta per cell (M). Values in bar graph are presented as means ± SEM (n = 5). Two-way ANOVA with Tukey’s multiple comparison test (n = 5): *p < 0.05, **p < 0.01, **p < 0.01, ***p < 0.001.

To test whether TET regulates autophagy pathway in lung fibrosis, we administered TET to TGF-β1-induced MLFs and evaluated the extent of autophagic flux. Conversion of MAP1LC3-I to MAP1LC3-II was decreased in fibroblasts by stimulation with TGF-β1, indicating impaired autophagy. The effects of TGF-β1 were reversed by co-administration of TET in a dose-dependent manner (Figures 3A,B). SQSTM1, a cargo receptor protein, is degraded upon the delivery of ubiquitinated proteins to autophagosomes (Mauvezin and Neufeld 2015). We found that inhibition of the autophagy flux with chloroquine (CQ, which blocks fusion of autophagosomes and lysosomes) “TET regulated SQSTM1 in both autophagy dependent and independent manner” following treatment with TET induced the cytosolic accumulation of the SQSTM1 protein (Figure 3G). This observation indicates that the SQSTM1 protein is regulated by TET partially in an autophagy dependent manner. However, the inhibition of autophagy initiation by 3 MA treatment also induced the accumulation of the SQSTM1 protein (Figure 3E). Based on the above results, we believe that the increase in SQSTM1 by TET is likely to occur at the transcription regulation, rather than just a consequence of autophagic inhibition. Further, we observed that mRNA level of SQSTM1 is significantly upregulated by TET through PCR experiment (Figure 3D), which could positively contribute to the TET-induced autophagic flux. It is indicated that TET regulated SQSTM1 in both autophagy dependent and independent manner. Similarly, Immunofluorescence results indicated that the TET-induced punctate staining of MAP1LC3B was significantly increased after treatment with CQ, and the effect of TET was suppressed by 3-MA in TGF-β1-treated fibroblasts. (Figures 3I,J). Transmission electron microscopy (TEM) further supported the induction of autophagy by TET in TGF-β1-stimulated fibroblasts (Figure 3K). To further demonstrate whether TET restores TGF-β1-induced impaired autophagy, a double tagged MAP1LC3 (mRFP-GFP) plasmid was used to examine autophagic flux. Notably, consistent with rapamycin (RAP, a representative autophagy promotor), TET treatment increased red fluorescence (red, autolysosomes), whereas CQ treatment accumulated yellow fluorescence (yellow, autophagosomes) in TGF-β1-induced MLFs (Figures 3L,M).

In order to validate the relevance of TET with apoptosis in lung fibrosis, we examined the expression of cleaved-caspase3. The results show that TET did not affect the expression of cleaved-caspase3 (Supplementary Figures S2A–E). Additionally, TET did not induce apoptosis as shown on flow cytometry and TUNEL assay (Supplementary Figures S2F–H). These findings indicated that TET did activate autophagic flux characterized by SQSTM1 accumulation.

### TET Activated SQSTM1-Mediated Selective Autophagy

**FIGURE 4:**
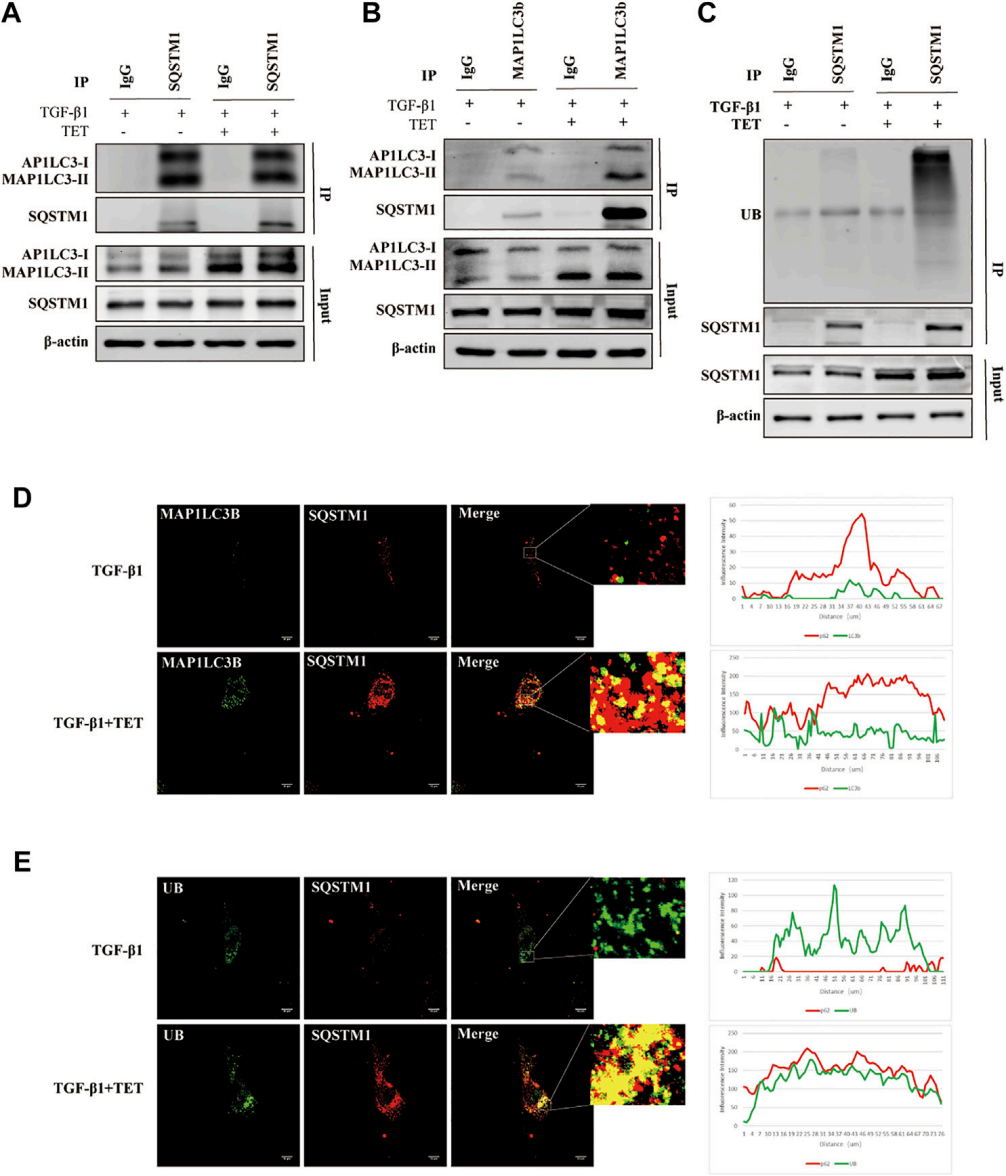
TET promotes the interaction of SQSTM1 with MAP1LC3B and with ubiquitinated protein. Primary MLFs were pretreated with TET or DMSO for 1 h and then subsequently stimulated with TGF-β1 for 24 h. **(A,B)** Co-immunoprecipitation (Co-IP) assays with SQSTM1 and MAP1LC3-I/II. Samples before (Input) and after (IP) immunopurification were analyzed by immunoblotting using SQSTM1 and MAP1LC3-I/II antibodies. **(C)** Co-IP assays with SQSTM1 and ubiquitinated protein. Samples were analyzed by immunoblotting using SQSTM1 and ubiquitin (UB) antibodies. **(D)** Confocal microscopy analysis of co-localization of MAP1LC3B and SQSTM1 in primary MLFs. The representative single optical sections and merge images are shown in the right panel. In these representative images, MAP1LC3B is visualized in green, SQSTM1 in red. Scale bars: 10 μm. **(E)** Confocal microscopy analysis of co-localization of UB and SQSTM1. Scale bars: 10 μm. UB is visualized in green, SQSTM1 in red.

The prevailing view is that ubiquitin-tagged misfolded proteins are assembled into aggregates by SQSTM1 (selective autophagy receptor), and the aggregates are then engulfed and degraded by autolysosomes (Svenning and Johansen, 2013). Interaction between selective autophagy receptors and MAP1LC3B is the molecular basis for selective autophagy. We next test whether TET induce autophagy in a SQSTM1 dependent manner. Our IP results show that TET treatment enhanced interaction of SQSTM1 and MAP1LC3B in TGF-β1-induced fibroblast (Figures 4A,B). Furthermore, the binding of SQSTM1 to ubiquitylated protein was significantly increased by TET (Figure 4C). Similarly, TET treatment efficiently enhanced the colocalization of SQSTM1 and MAP1LC3B and induced the SQSTM1-recruited cargos to autophagosomes (Figures 4D,E). Together, TET positively regulated selective autophagy via targeting SQSTM1.

### NRF2 Regulated SQSTM1 Transcription Involved in TET-Induced Selective Autophagy

**FIGURE 5:**
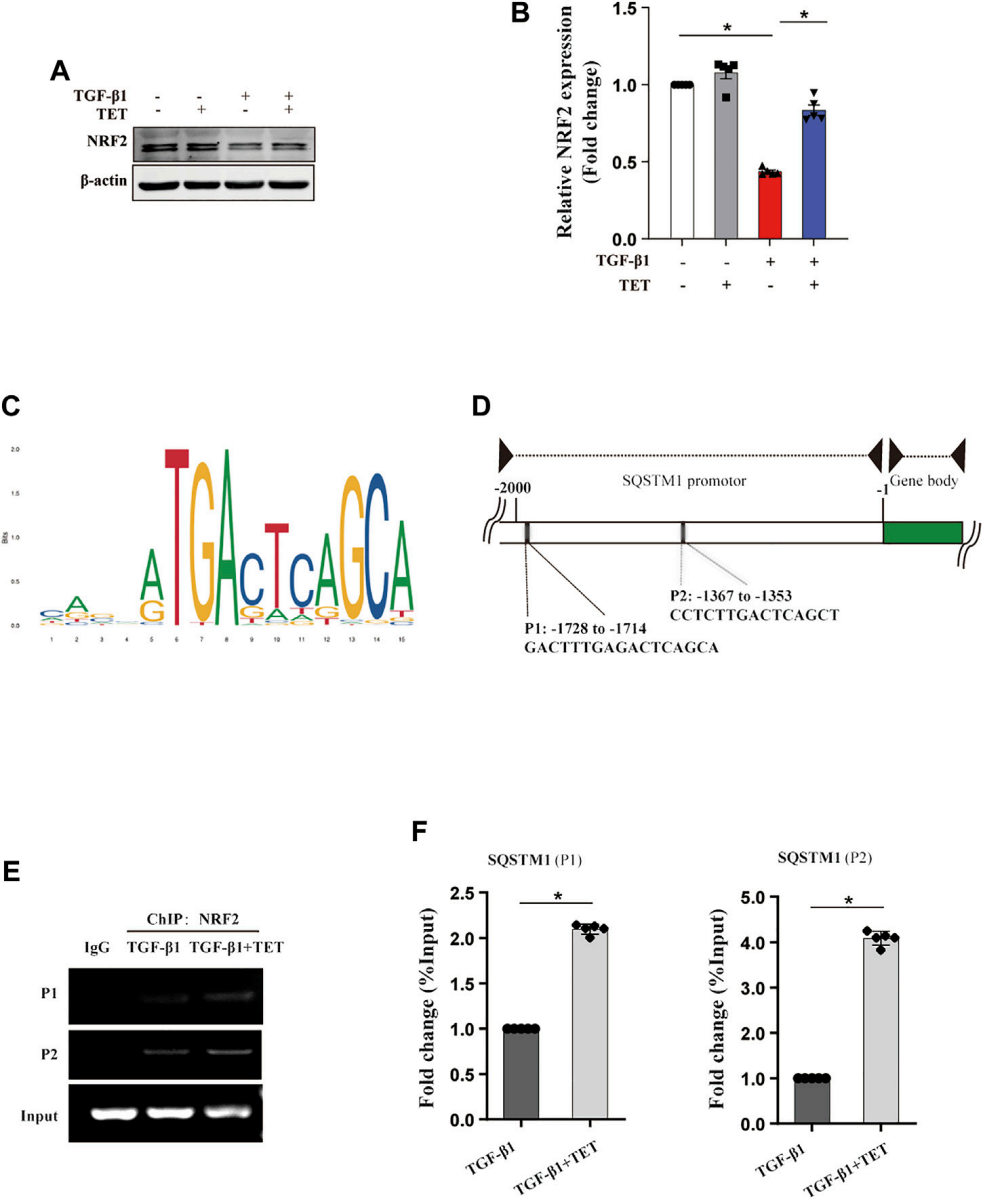
TET regulates the transcription of SQSTM1 through NRF2. Primary MLFs were treated with TGF-β1 (0 or 10 ng/ml) for 24 h in the presence/absence of TET (4 μM). **(A,B)** Immunoblot assays **(A)** and densitometric analysis **(B)** showed the expression of NRF2. **(C)** Identification of SQSTM1 promoter-containing genes in the mouse genome by the FIMO software tool. **(D)** Schematic representation of the SQSTM1 promotor region. PCR primers used for amplification (P1 and P2) are shown in the underneath schematic representation. **(E)** ChIP-PCR analysis to showing the association of NRF2 with the SQSTM1 promotor in MLFs. *RT*-*PCR* products were resolved by agarose gel electrophoresis. **(F)** *Statistical analysis* was obtained. Values in bar graph are presented as means ± SEM (n = 5). Two-way ANOVA followed by Tukey’s multiple comparisons test was used for statistical analysis of **(B)** and Student’s t-test was used for **(F)**. *p < 0.05.

It has been demonstrated that both protein and mRNA expression of SQSTM1 were increased by TET, partially independent of autophagy pathway. We aim to investigate how TET regulates SQSTM1. Previous research suggested that transcription factor NRF2 can bind to promoter of SQSTM1, leading to promoted SQSTM1 transcription. We found that expression of NRF2 was decreased by TGF-β1, but increased by treatment with TET in fibroblasts (Figures 5A,B). To further confirm that SQSTM1 is regulated by NRF2, we performed ChIP for endogenous NRF2 in primary MLFs and analyzed the enrichment of NRF2 at the transcription start site (TSS). Firstly, using NCBI website and JASPAR programs, we analyzed the SQSTM1 promoter sequence to predict transcription factor binding sites (Figures 5C,D). Our data showed that TET increased enrichment of NRF2 at binding sites of SQSTM1 in TGF-β1-stimulated fibroblasts (Figures 5E,F).

### TET Activated SQSTM1-Mediated Selective Autophagy via Rheb-mTOR Signaling

**FIGURE 6:**
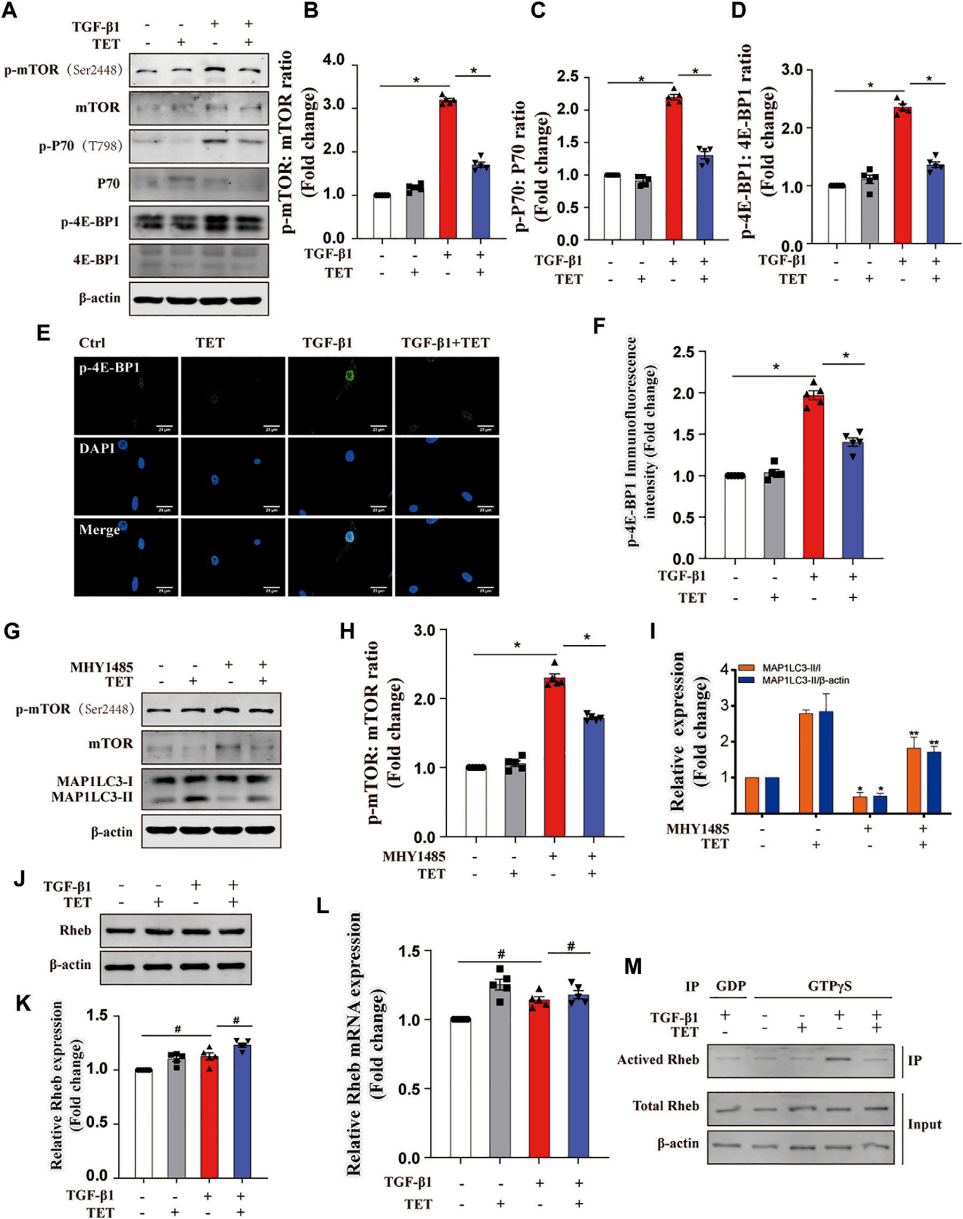
TET activates autophagy by inhibiting Rheb/mTORC1 signaling. **(A–D)** Effect of TET on mTOR activation. Immunoblot **(A)** and quantitative analysis **(B–D)** showed the expression level of mTOR phosphorylation (p-mTOR), *mTOR* downstream substrates (p-P70, p-4E-BP1). **(E,F)** Representative images of immunofluorescence monitored by confocal microscopy **(J)** and quantitative analysis **(K)** showed the expression of p-4E-BP1. Green staining is p-4E-BP1, blue staining indicates nuclei. Scale bars: 20 μm. **(G–I)** Effect of mTOR activation on autophagy regulation. Primary MLFs were cotreated with TET (4 μM) and MHY1485 (5 μM) for 24 h. Immunoblot (G) and quantitative analysis **(H–I)** showed the expression of autophagy markers (MAP1LC3-I/II, SQSTM1), mTOR activation (mTOR, p-mTOR). **(J,K)** Effect of TET on the expression of Rheb protein. Representative immunoblot analysis **(J)** and quantitative analysis **(K)** show the expression of Rheb protein. **(L)** Representative of qPCR analysis showed the mRNA expression of Rheb. **(M)** Effect of TET on Rheb activation. Co-immunoprecipitation was performed using agarose beads conjugated to an antibody directed against the GTPγS and GDP. Immunoblot analysis was performed to check for protein expression of activated Rheb (Rheb/GTP), total Rheb. Total Rheb includes the inactive GDP-Bound form (GDP) and activated Rheb (Rheb/GTP). Values in bar graph are presented as means ± SEM (n = 5). Two-way ANOVA with Tukey’s multiple comparison test (n = 5). *p < 0.05.

The mTOR kinase is a key regulator of autophagy induction (Hu et al., 2015). As our results show, TET significantly suppressed TGF-β1-induced mTOR activation and phosphorylation of P70 and 4E-BP1 (Figures 6A–D). Similarly, 4E-BP1 inhibition by TET was confirmed by immunofluorescent staining (Figures 6E,F). To investigate the role of mTOR during TET-induced autophagy, fibroblasts were treated with MHY1485, an activator of mTOR, after TET stimulation. Importantly, TET enhanced conversion of MAP1LC3-I to MAP1LC3-II, and this effect was reduced by MHY1485 (Figures 6G–I). These results suggest that TET-induced autophagy is related to the inhibition of mTOR signaling pathway.

Previous studies suggest mTOR activation is regulated by Rheb, a Ras-like small guanosine triphosphatase (GTPase) (Goordan et al., 2011; Narita et al., 2011). However, both the total Rheb protein and mRNA expression was not reduced by TET (Figures 6J–L). We next determined the Rheb activity by Co-IP. Our results showed that TET reduces Rheb activity in TGF-β1-stimulated fibroblasts (Figure 6M), suggesting TET negatively regulates Rheb activity but not protein or mRNA expression. Taken together, TET activated autophagy through Rheb-mTOR signaling.

### TET Attenuated TGF-β1-Induced Myofibroblasts Differentiation and Proliferation by Inducing Autophagy *In Vitro*

**FIGURE 7:**
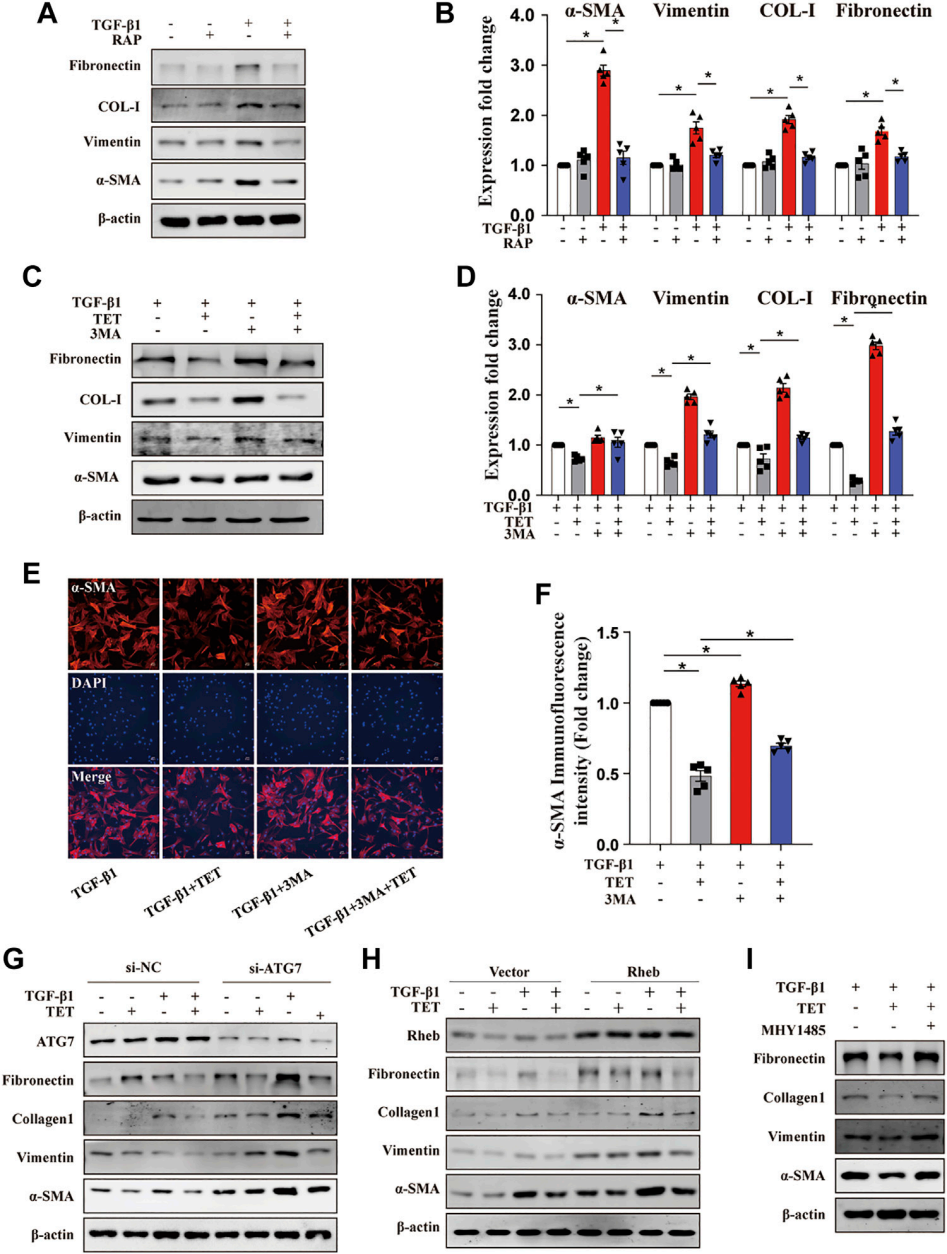
TET improves pulmonary fibrosis by activating autophagy through Rheb/mTOR in MLFs. Primary MLFs were treated with TGF-β1 (0 or 10 ng/ml) for 24 h in the presence/absence of TET (4 μM), 3 MA (500 nM), RAP (50 nM), or MHY1485 (a small molecule mTOR activator, 5 μM). **(A,B)** Representative immunoblot analysis **(A)** and quantitative analysis **(B)** showed the expression of fibrosis-associated proteins. **(C,D)** The effect of autophagy on TGF-β1-induced primary MLFs. Immunoblot **(C)** and quantitative analysis **(D)** showed the expression of ECM deposition, myofibroblasts transdifferentiation markers. **(E,F)** Confocal microscopy images **(E)** and relative fluorescence intensity of α-SMA **(F)**. α-SMA was visualized in green, DAPI-stained nuclei in blue. Scale bars: 50 μm. **(G)** MLFs transfected with nonspecific nonsilencing negative siRNA control (*si*-*NC*) or ATG7 small interfering *RNA* (*si*-ATG7) for 48 h were treated with TET and TGF-β1. Representative immunoblot analysis showed the expression of fibrosis-associated proteins. **(H)** Cells transfected with the empty vector plasmid (vector) or Rheb overexpression plasmid (Rheb) for 48 h and then incubated with TET and TGF-β1. Immunoblot of fibrosis-associated proteins. **(I)** TET-attenuated fibrosis was associated with mTOR activation. Immunoblot showed the expression of fibrosis-associated proteins. Values in bar graph are presented as means ± SEM (n = 5). Two-way ANOVA with Tukey’s multiple comparison test (n = 5). *p < 0.05.

To further examine whether autophagy induction contributes to TET-mediated protective effects in lung fibrosis, we performed pharmacological and transgenic approaches to inhibit autophagy and detected fibrotic markers. The addition of Rapamycin (autophagy inducer as a positive control) downregulated expression of fibrotic markers in fibroblasts stimulated by TGF-β1 (Figures 7A,B). These results supported that autophagy inducers can attenuate lung fibrosis. We observed that fibronectin, collagen type Ⅰ, vimentin, and α-SMA were all decreased by TET in TGF-β1-induced fibroblasts, and this effect was blocked by 3 MA (Figures 7C–F). Furthermore, silencing of ATG7 caused efficient downregulation of ATG7 protein expression compared with a non-silencing control and markedly inhibited TET-decreased expression of fibrotic markers in fibroblasts (Figure 7G). To test whether Rheb/mTOR signaling controls fibroblasts transdifferentiation, Rheb was overexpressed in fibroblasts. The effects of TET on suppressing fibrotic markers induced by TGF-β 1 was lost when Rheb is overexpressed (Figure 7H). Additionally, TET reduced TGF-β1-stimulated fibrotic expression, which was blocked by MHY1485, a small molecule activator of mTOR (Figure 7I). Taken together, TET attenuates lung fibrosis through inducing autophagy.

### Type I Collagen Is Degraded by TET-Induced Autophagy

**FIGURE 8:**
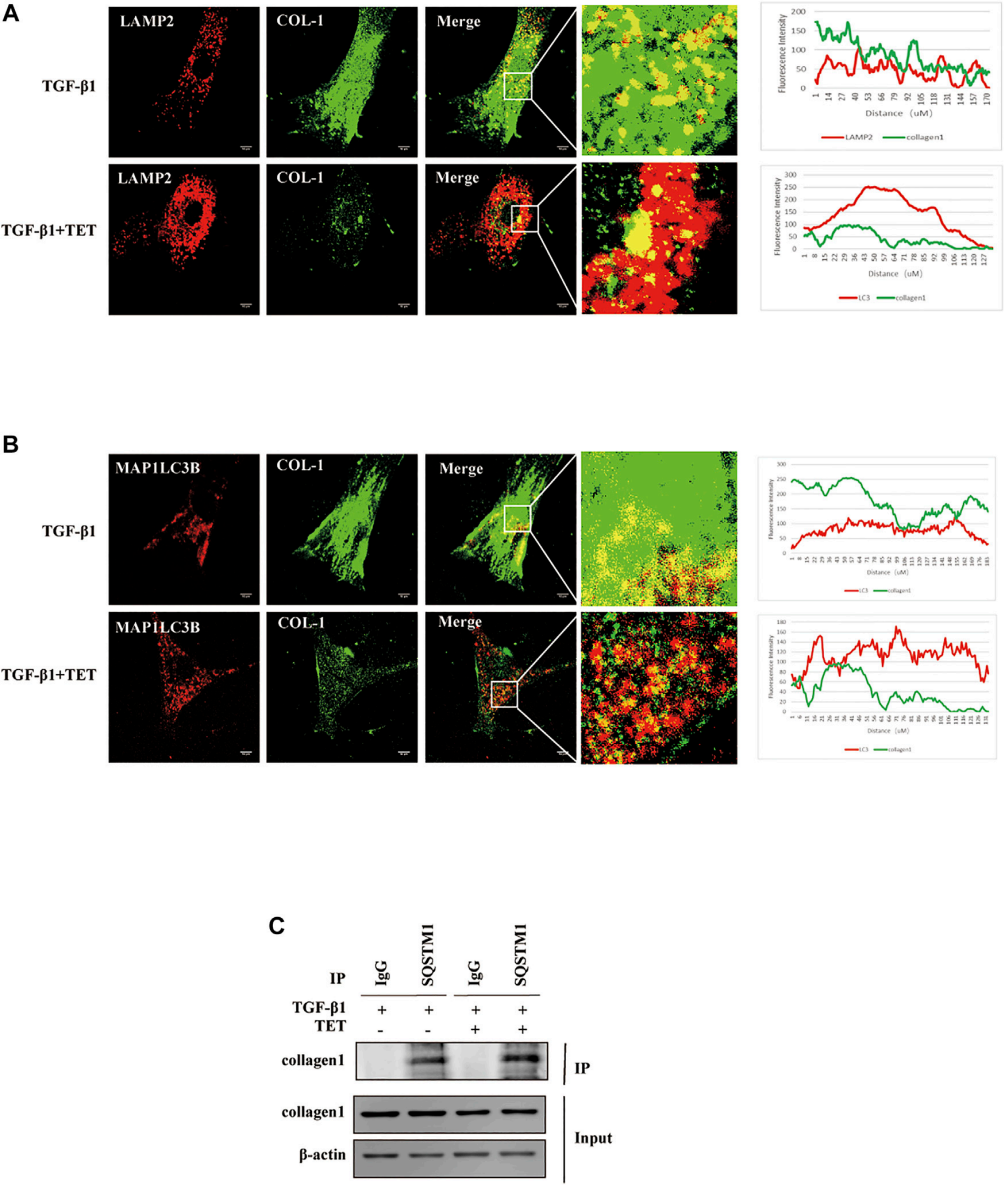
Col-I is degraded by autophagy. Primary MLFs treated with DMSO or TET for 1 h and then subsequently simulated with TGF-β1 for an additional 24 h. **(A)** Confocal microscopy analysis of colocalization of LAMP2 (lysosomal markers, red) and COL-I (green). The representative single optical sections and merge images are shown. Scale bar: 10 μm. **(B)** Confocal microscopy analysis of colocalization of MAP1LC3B (red) and COL-I (green). The representative single optical sections and merge images are shown. Scale bar: 10 μM. **(C)** Co-immunoprecipitation (Co-IP) assays with SQSTM1 and COL-I. Samples before (Input) and after (IP) immunopurification were analyzed by immunoblotting.

As shown before, TET presented a significant decrease in the steady-state levels of collagen inducing by TGF-β 1. However, the mechanism is unknown. Since previous data indicated that intracellular Col-I could degrade by lysosome (Sosulski et al., 2015) we hypothesized that Col-I is degraded by autophagy. The addition of TET increased the appearance of lysosomes and the colocalization of Col-I and lysosomes in TGF-β1-stimulated fibroblasts, indicating lysosomal degradation of Col-I (Figure 8A). Similarly, there are more endogenous Col-I colocalized with autophagosomes in cells treated with TET after TGF-β 1 stimulation (Figure 8B), further confirming that Col-I is degraded by autophagy. Interestingly, TET improved interaction of Col-I and SQSTM1 in TGF-β 1-induced fibroblasts (Figure 8C). These results suggest that Col-I is delivered to the lysosome *via* SQSTM1 for degradation during TET-induced autophagy.

### TET Attenuated Lung Fibrosis in bleomycin-Induced Mouse Models Through Activating Autophagy

**FIGURE 9:**
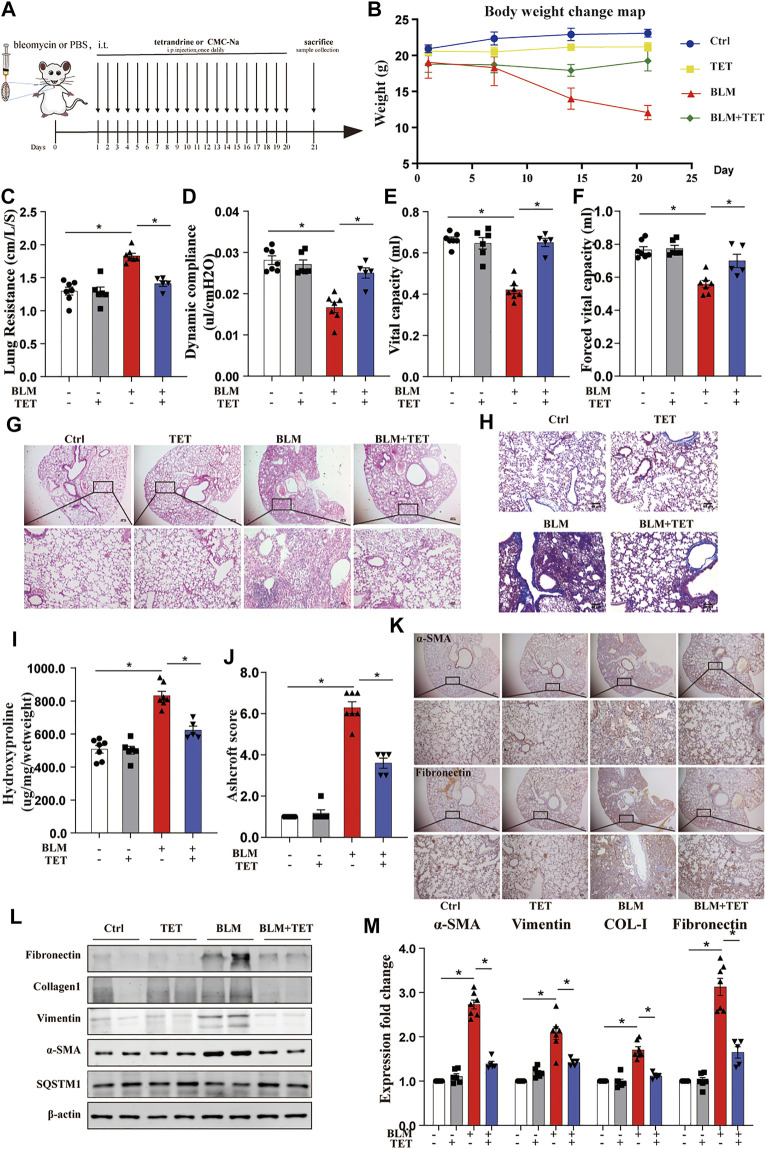
TET treatment protects against pulmonary fibrosis induced by bleomycin. Mice, were treated prophylactically with either vehicle (sodium carboxymethyl cellulose, i. p, daily) or TET (20 mg kg^−1^·d^−1^, i.p.) starting on Day 1 after receiving a single intratracheal administration of BLM. The control group received intratracheal PBS. **(A)** Schematic diagram of the time course of TET treatment in a mouse model of BLM-induced pulmonary fibrosis. **(B)** Changes in body weight were presented relative to the initial weight. **(C-F)** The pulmonary function parameters were measured by pulmonary function test. **(G)** Lung tissue was sectioned at day 21 and performed HE staining. Scale bar of top images: 100 μm, below images: 40 μm. **(H)** Lung tissues were stain with Masson trichrome staining. Scale bars: 40 μm. **(I)** Ashcroft scores were analyzed. **(J)** Hydroxyproline (HYP) expression of each group by hydroxyproline assay. **(K)** The protein expression of α-SMA (left images) and fibronectin (right images) in lung sample were examined by immunohistochemical staining. Scale bar of top images: 100 μm. Scale bar of below images: 40 μm. **(L-M)** Lung tissues were treated as described in (A) and subjected to immunoblots of fibrosis and autophagy-associated proteins(L) and densitometric analysis was obtained **(M)**. The data were presented as the means ± SEM (n ≥ 5). Two-way ANOVA followed by Dunnett’s multiple comparisons test was used for statistical analysis. *p < 0.05.

TET is a safe and widely used agent for silicosis and has therapeutic potential to restore cell metabolic homeostasis (Zhang et al., 2018). We explored whether TET can accelerate the resolution of fibrosis in the bleomycin (BLM)-induced lung fibrosis model. We treated mice with BLM at day 0 and started daily TET treatment from day 1–21 after BLM administration to examined the preventative effects of TET on pulmonary fibrosis (Figure 9A). Notably, TET attenuated BLM-induced mice body weight loss and impaired pulmonary function (Figures 9B–F). TET therapy significantly alleviated pulmonary fibrosis including improvement of the disordered lung structure and reduction of collagen deposition (Figures 9G–J). Histology and immunohistochemistry showed diminished amounts of collagen and α -SMA were seen in TET treatment group compared with BLM group (Figure 9K). Consistent with the histological analysis, immunoblotting showed that TET blocked the increase of extracellular matrix (ECM) deposition and α-SMA protein in BLM-challenged mice (Figures 9L–M). Importantly, these effects of TET are accompanied with autophagy activation (Figure 9L).

We also examined the therapeutic effects of TET on pulmonary fibrosis (Supplementary Figure S4A). These results demonstrated significant reduction in several fibrosis-related changes, including total lung hydroxyproline, histologic change, ECM deposition, and weight loss (Supplementary Figure S4B–F). These data indicated that TET activated autophagy to blunt bleomycin-induced lung fibrosis, suggesting a further potential therapeutic effect of TET on pulmonary fibrosis.

## Discussion

**FIGURE 10:**
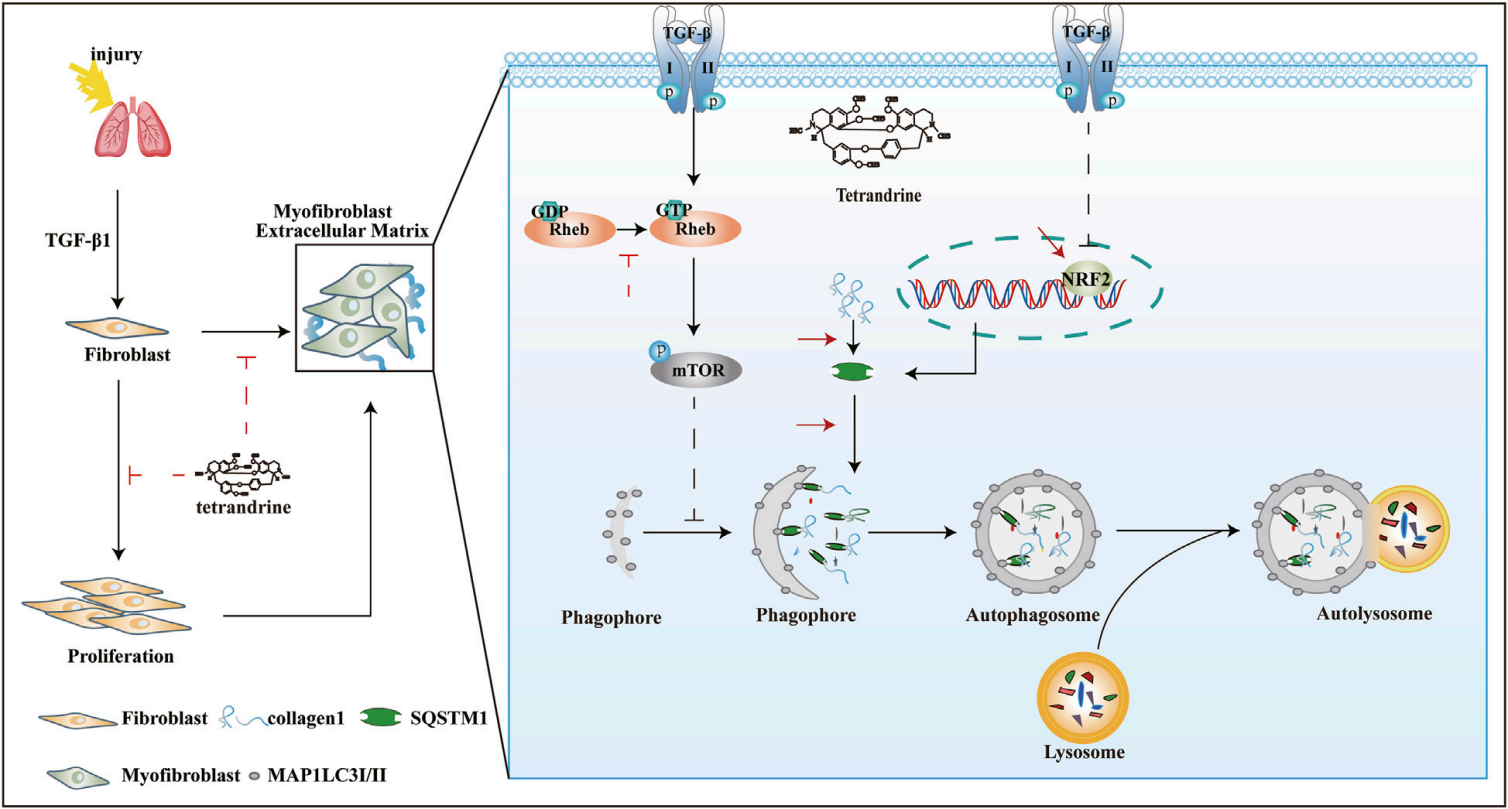
Underlying mechanism of TET against pulmonary fibrosis through SQSTM1-mediated activation of autophagy. See the text for details.

TET, originally isolated from Chinese herbs but now produced synthetically, has been tested for clinical trials and found to be effective against silicosis and lung cancer (Bhagya and Chandrashekar 2016). However, whether TET has anti-fibrotic activity and its potential mechanisms have not been systematically evaluated. In this study, we provide evidences that TET can resolve pulmonary fibrosis through inhibiting myofibroblast differentiation, proliferation, and ECM deposition. We found that TET can enhance the interaction of SQSTM1 with MAP1LC3-II and ubiquitinated proteins due to NRF2-mediated SQSTM1 transcription and Rheb-mTOR signaling activation, thus dramatically inducing SQSTM1-selective autophagy and directly leading to Col-I degradation in lysosome (Figure 10). This is the first report that showed the potential new mechanisms involved in TET-induced selective autophagy in the context of lung fibrosis.

TET therapy significantly attenuated experimental lung injury and produced similar therapeutic results in animal models of cardiac and liver injury. In a previous study, inhalation of TET could alleviate pulmonary fibrosis in a mouse model (Su et al., 2020). However, its precise role and its therapeutic utility in IPF remains unclear. Our results further explore the therapeutic role of TET on lung fibrosis. TET therapy effectively improved pulmonary function and decreased fibrotic markers in two mouse models. Specially, our data suggest that TET may play a role in reversing established pulmonary fibrosis. This is indicated by our observation that delayed administration of TET at day 8 after BLM-induced pulmonary fibrosis inhibits the procession of pulmonary fibrosis. Similarly, TET inhibits TGF-β1-induced fibroblast differentiation, proliferation, and ECM deposition *in vitro*. Impressively, TET therapy did not induce apoptosis, in contrast with previous studies (Liu et al., 2011), because the TET dose we used is not high to 30 μM. We suggest a more safe and effective concentration in lung fibrosis.

Network pharmacology studies emphasize the paradigm shift from “one target, one drug” to “network target, multicomponent therapeutics,” highlighting a holistic thinking also shared by traditional Chinese medicine (TCM) (Li et al., 2014). Through network pharmacology analysis we found that the anti-fibrotic effect of TET may be related to autophagy. Previous studies using lung biopsies from IPF patients reported a diminution of autophagy (Patel et al., 2012; Araya et al., 2013) and some from animal model illustrated that TGF-β1 could mediate dysfunction of the autophagy response during lung fibrosis (Sosulski et al., 2015)*.* Herein, we impart an appreciation for TET-mediated regulation of the autophagic response in TGF-β1-induced MLFs. Our findings demonstrated that TET may restrain differentiation of lung fibroblasts and ECM deposition through increasing autophagy flux. Furthermore, TET increased autophagy flux, MAP1LC3-Ⅱ/SQSTM1 dependent ubiquitinated protein recycling, and lysosome degradation of Col-I. This study connects autophagy and protein homeostasis to lung fibrosis. Nevertheless, the redundancy of mechanism of TET that regulate lysosome homeostasis needs to be investigated, as protein metabolism is a complex process that involves multiple interacting signaling pathways (Araya and Nishimura 2010).

SQSTM1 serves as multifunctional regulator of cell signaling involved in selective autophagy (Svenning and Johansen, 2013; Lamark et al., 2017)*.* Both MAP1LC3-Ⅱ and SQSTM1 are required to maintain autophagy flux that promoted recruitment of SQSTM1-associated ubiquitinated proteins towards autophagosome-lysosome degradation. Interestingly, TET treatment significantly increases protein levels of SQSTM1 and MAP1LC3-Ⅱ in TGF-β1-induced MLFs. It is important to confirm that the SQSTM1 mRNA level has not changed if the SQSTM1 protein level is used an indicator of autophagy flux. We provide evidence that TET dramatically increases mRNA expression of SQSTM1 in TGF-β1-induced MLFs. In addition, CQ, a lysosome inhibitor, can further increase protein levels of SQSTM1 at present of TET. This observation indicates that the SQSTM1 regulation is likely to occur at the level of transcription rather than due to the SQSTM1 protein accumulation as a consequence of autophagic inhibition. The increase in SQSTM1 expression by TET seemed to be autophagy-independent manner, but could positively contribute to the TET-induced autophagic flux. Previous studies suggest that TGF-β1-induced impaired autophagy is a critical pathogenesis of IPF, major in SQSTM1 gene repression. Emerging studies have found that NRF2 binds to the antioxidant response element (ARE) of the SQSTM1 promoter, leading to increase SQSTM1 transcription (Liu et al., 2007).

NRF2, a critical transcription factor, has a major anti-oxidant and anti-inflammatory effect. During stress conditions, NRF2 dissociates from Keap1 and translocates into the nucleus to regulate target genes through binding to ARE in their promoters (Hayes and McMahon 2009; Iso et al., 2016). However, the interaction of NRF2 and SQSTM1 is not confirmed in lung fibrosis. We show TET enhances the association between NRF2 and SQSTM1 and executes its anti-fibrosis effect via NRF2-SQSTM1pathway. Besides, defects in SQSTM1 may contribute to the deregulation in NRF2 activity seen in myofibroblasts and pulmonary fibrosis (Ichimura et al., 2013; Bian et al., 2018). Thus, up-regulation of SQSTM1 by TET may provide dual protection to TGF-β1-induced MLFs through facilitating both selective autophagy and the NRF2-mediated antioxidant response. Moreover, further investigations are required to clarify the involvement of NRF2 in the SQSTM1 transcription.

The Ras homolog enriched in the brain gene (Rheb) is ubiquitously expressed in mammalian cells and encodes proteins that play an important role in regulating cell growth and survival (Bai et al., 2007). Rheb exists either in an active GTP-bound state or an inactive GDP-bound state and only Rheb-GTP activates the rapamycin complex 1 (TORC1) (Goordan et al., 2011). Rheb/mTORC1 signaling plays a critical role for fibroblast activation in kidney fibrosis (Jiang et al., 2013). Our results show that TET inhibits the activity of Rheb by stimulating the conversion of Rheb-GTP to Rheb-GDP to repress mTOR signaling. Rheb activates mTOR signaling, including increased p-p70S6K and p-4E-BP1 (Huang and Manning 2008). Some studies provide strong support that mTORC1 signaling induces canonical Smad activation via 4E-BP1 phosphorylation which strongly promotes the profibrotic effect of TGF-β1 (Jiang et al., 2013). Indeed, TET inhibits mTORC1- 4E-BP1 signaling in TGF-β1-induced MLFs. Based on above data, we demonstrate that TET decreased collagen deposition induced by TGF-β1, partially through mTORC1/4E-BP1 signaling.

On the other hand, mTORC1 has been well established as the key negative regulator of autophagy, *via* suppression of the ULK1 complex at the initiation of autophagy (Yu et al., 2010). In this section, our data indicate TET can induce autophagy by inhibiting mTORC1 signaling pathway. Although mTORC1 is inactivated during autophagy initiation, it can be reactivated when energy supplies through the degradation of autolysosomal products at the end of autophagy flux. Interestingly, we found that TET can significantly increase the number of lysosomes which might be because of reactivated mTORC1. Its reaction is required for the reformation of functional lysosomes (Yu et al., 2010)*.* Previous studies have been suggested that mTORC1 directly phosphorylates TFEB, a master transcriptional regulator of lysosomal and autophagy genes (Settembre et al., 2011). Taken together, TET induces autophagy by inactivating mTORC1 to supply more energy, then might reactivate mTORC1 in time-dependent manner to improve lysosome cycle. Nevertheless, further experiments will need to be performed to support this assumption.

Additionally, intracellular degradation of Col-I via autophagy indicates a critical role of autophagy in collagen homeostasis (Kim et al., 2012). We provide evidence TET improves co-localization CoI-I with LAMP2 and MAP1LC3-II. Among these, we suggest TET reduced Col-I accumulation by dragging Col-1-LC3 complex into the lysosome for degradation. Therefore, our study provides initial evidence that TET -mediated degradation of Col-I through autophagy was partially resistance to pulmonary fibrosis.

In summary, TET, a monomeric component of traditional Chinese medicine, ameliorates BLM-induced experimental lung fibrosis. In addition, our results showed that TET exerts antifibrotic effects via NRF2/SQSTM1 signaling and Rheb/mTORC1 pathway mediated autophagy. Furthermore, TET can promote SQSTM1 transcription by NRF2. This study shows a novel mechanism that NRF2 and SQSTM1 play fundamental roles through regulating autophagy in lung fibrosis. Based on these findings, TET should be considered as a therapeutic option for IPF patients.

## Data Availability

The original contributions presented in the study are included in the article/Supplementary Material, and further inquiries can be directed to the corresponding authors.
