# Supplementary material for: Tetrandrine Modulates Rheb-mTOR Signaling-Mediated Selective Autophagy and Protects Pulmonary Fibrosis
Source: Front Pharmacol. 2021 Nov 22;12:739220. doi: 10.3389/fphar.2021.739220 (PMC8645995; doi:10.3389/fphar.2021.739220)
Supplement: Supplementary file 1 [file DataSheet1.PDF]

Supplementary materials and methods

Detection of apoptosis and cell-cycle fractions by flow cytometry

Apoptosis was determined by annexin V–FITC and propidium iodide double staining according to the manufacturer's instructions (BD Biosciences). Cell-cycle fractions were determined by propidium iodide nuclear staining. Briefly, cells were harvested, washed in PBS, fixed in 70% ethanol for 30 minutes on ice, and incubated in propidium iodide solution (20 μg/mL propidium iodide, 0.2 mg/mL RNAse A in PBS) for 30 minutes at 37°C. Data were collected on a FACSCalibur flow cytometer (BD Biosciences) and analyzed with FlowJo Version 7.5.5 software (TreeStar).

Apoptosis assay

Apoptosis was assessed using the terminal deoxynucleotidyl transferase–mediated dUTP nick-end labeling (TUNEL) kit (PharMingen) according to the manufacturer's instructions. Annexin V staining was performed using the TACS annexin V–FITC kit from Dojindo (Japan) following the manufacturer's protocol.

Supplementary Table 1 siRNA and plasmid sequences list

**Gene**	**Sequence(5'→3')**
**si ATG7 list2**	**F: GAGGCUGGUAAGAACAGUATT**<br>**R: UACUGUUCUUACCAGCCUCTT**
**si ATG7 list2**	**F: CAGACAAGAAGCUCCUUCUTT**<br>**R: AGAAGGAGCUUCUUGUCUGTT**
**si ATG7 list3**	**F: CUGAGAGCAUCCCUCUAAUTT**<br>**R: AUUAGAGGGAUGCUCUCAGTT**

Supplementary Figures

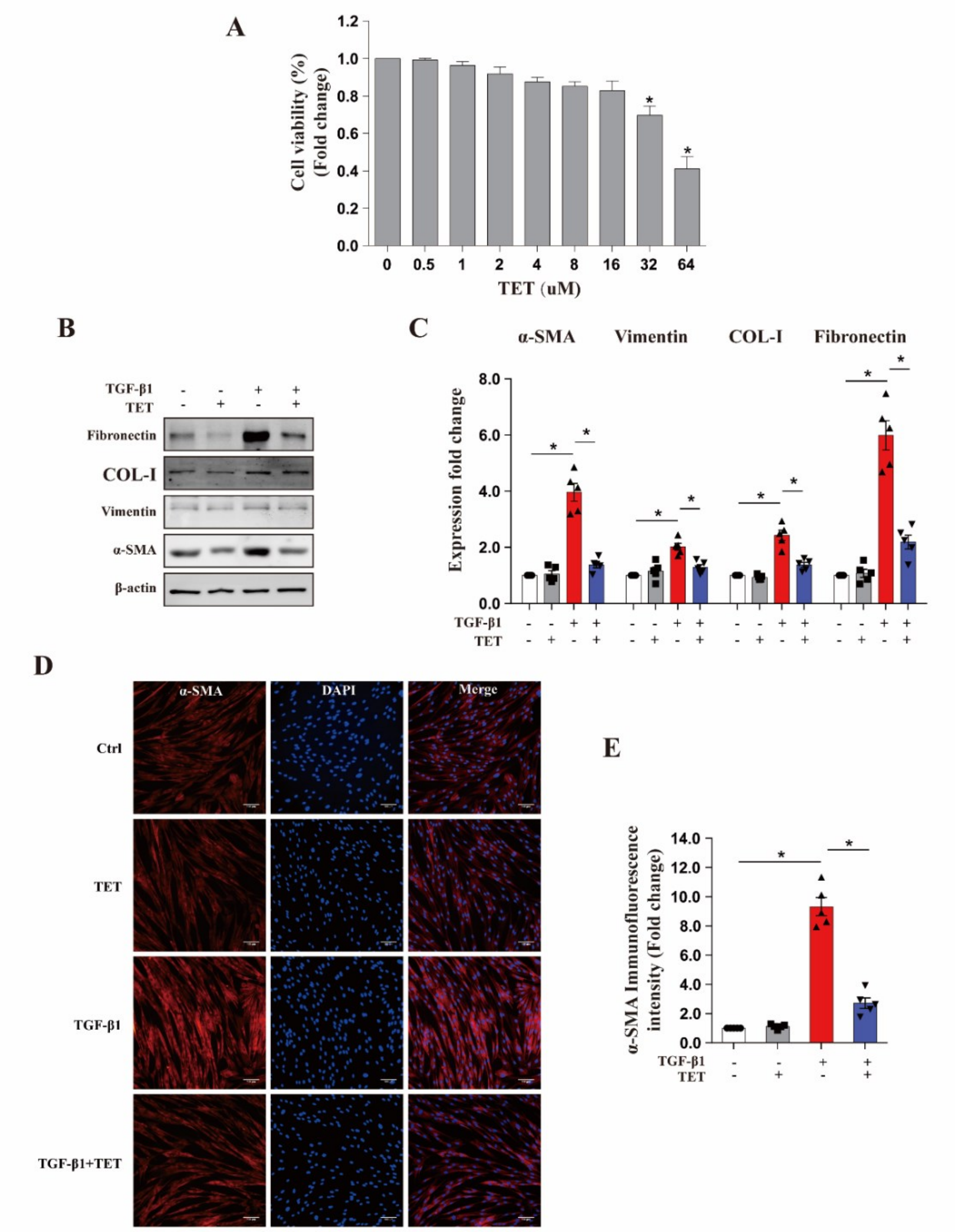

Supplementary Figure 1. Tetrandrine suppresses TGF-β1-induced myofibroblasts differentiation and ECM deposition in IMR90. (***A***) IMR90 were incubated with various indicated concentrations of TET for 24 h and subjected to CCK8 assay to assess cell viability. One-way ANOVA with Dunnett's multiple comparison test: *p < 0.05 comparison to TET=0 μM group value. **(*B* and *C*)** IMR90 were pretreated with DMSO or TET (4 μM) for 1 h and then subsequently incubated with or without TGF-β1 (10 ng/ml) for 24 h. Immunoblot analysis (B) and quantitative analysis (C) showed TET the expression of fibrosis-associated proteins in IMR90. **(*D* and *E*)** Representative Immunofluorescence monitored by confocal microscopy (D) and quantitative analysis (E) showed the expression of α-SMA (red) in IMR90. Blue staining indicates nuclei Scale bars: 100 μm. *P* values were determined by Two-way ANOVA with Tukey's multiple comparison test: *$p < 0.05$.

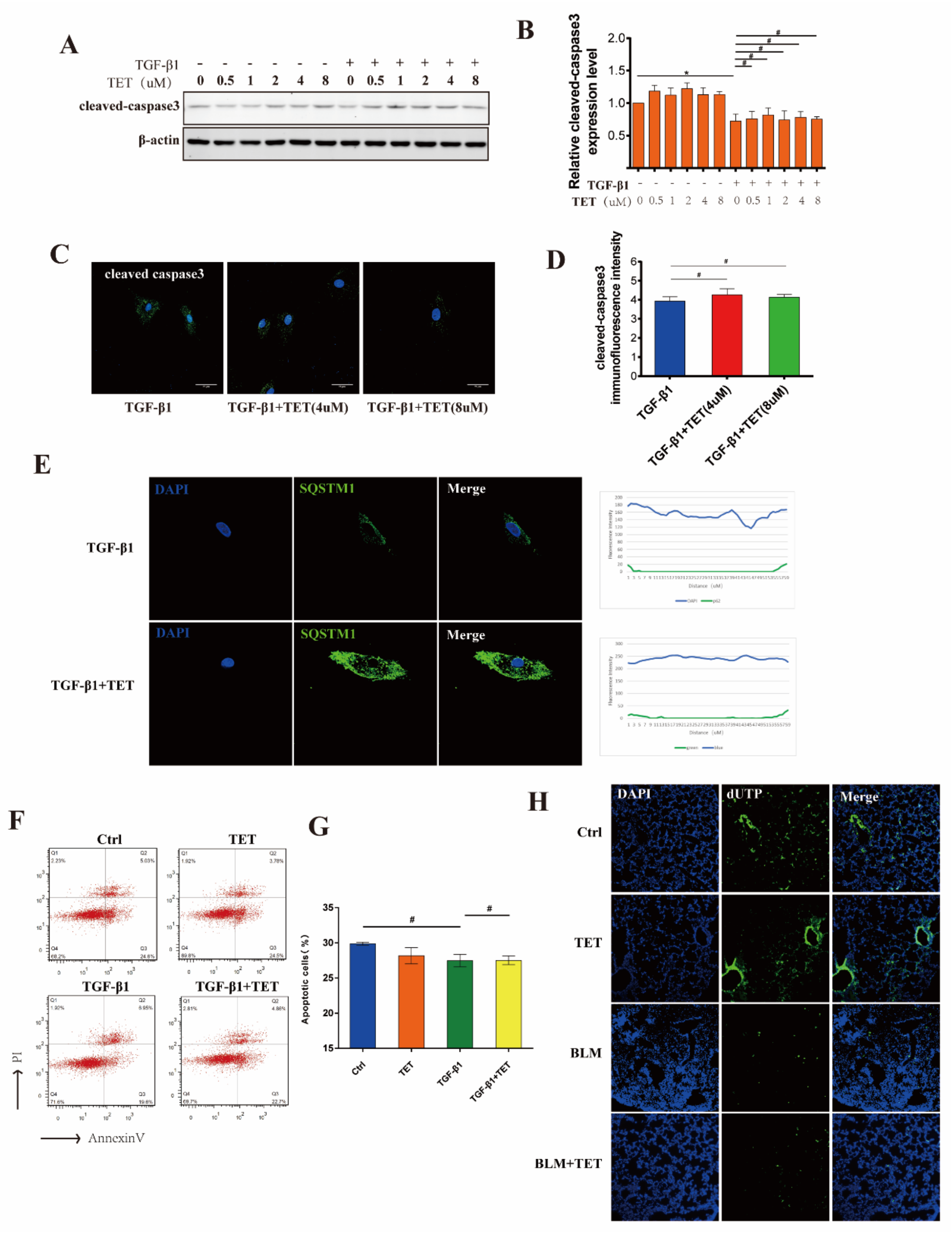

Supplementary Figure 2. TET has no effect on apoptosis. (***A-B***) Primary MLFs were pretreated with TET for indicated concentrations for 1 h and then subsequently stimulated with TGF-β1 for 24 h. Immunoblot analysis (A) and quantitative analysis (B) showed the expression of apoptosis (cleaved caspase 3) markers. Values in bar graph are presented as means ± SEM (n=5). Two-way ANOVA: **P* < 0.05, ***P* < 0.01, versus the control group; #*p* < 0.05, ##*p* < 0.01, versus the TGF-β1 group. (***C* and *D***) Immunofluorescence (C) and quantitative analysis (D) showed the expression of apoptosis marker (cleaved caspase 3). Green staining is cleaved caspase 3, blue staining indicates nuclei. cleaved caspase 3 puncta was monitored by confocal microscopy. Scale bars: 20 μm. (***E***) The effect of TET on SQSTM1 nuclear

translocation in vitro. Representative images of confocal fluorescence (left panels) and quantitative analysis (right panels) showed intracellular localization of the SQSTM1 protein. Green staining is SQSTM1, blue staining indicates nuclei. Scale bars: 20 μm. **(*F* and *G*)** FITC–Annexin V apoptosis detection with a flow cytometer (F) and the percentage of apoptotic cells were quantitated (G). **(*H*)** The apoptosis-positive cells at pulmonary tissue by TUNEL staining. Scale bar: 100 μm. Values in bar graph are presented as means ± SEM (n=5). *P* values were determined by two-way ANOVA followed by Tukey's multiple comparisons test. #*p*: no significant.

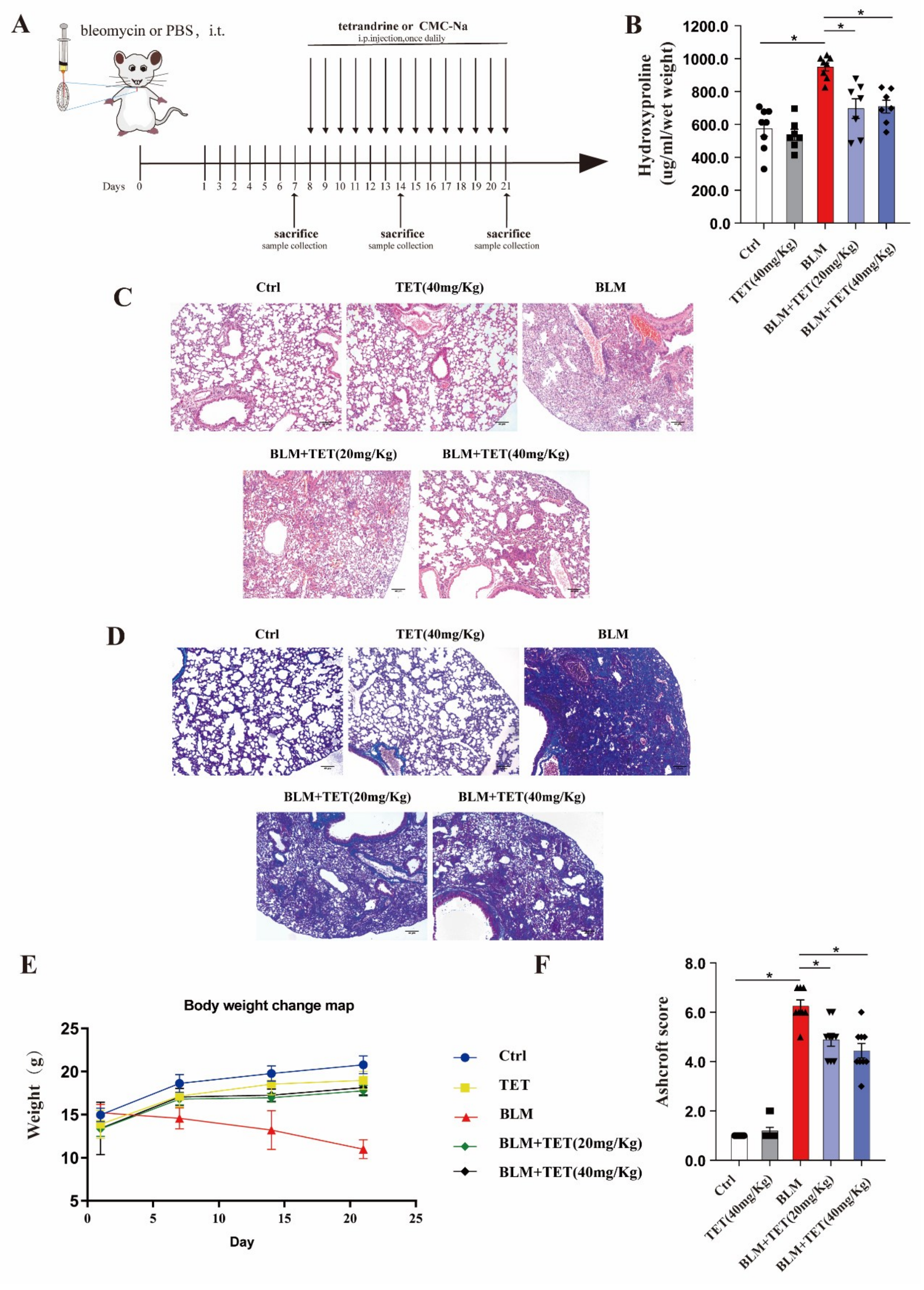

Supplementary Figure 3. Therapeutic effects of TET on bleomycin-challenged pulmonary fibrosis. Mice, were treated prophylactically with either vehicle (sodium carboxymethyl cellulose, i.p., daily) or tetrandrine (TET, 20 or 40 mg·kg-1·d-1, i.p.) starting on Day 8 after receiving a single intratracheal administration of BLM. The control group received intratracheal PBS. (***A***) Schematic diagram of the time course of TET treatment in a mouse model of BLM-induced pulmonary fibrosis. (***B***)

Hydroxyproline (HYP) expression of each group by hydroxyproline assay. ***(C)*** Lung tissue mice was performed HE staining. Scale bar: 40 μm. ***(D)*** Lung tissues were stain with Masson trichrome staining. Scale bar: 40 μm. ***(E)*** Changes in body weight are presented relative to the initial weight. ***(F)*** Ashcroft scores were calculated as described in the methods. The data were presented as the means ± SEM (n≥5). Two-way ANOVA followed by Dunnett's multiple comparisons test was used for statistical analysis. *$P <$ 0.05.
